# The efficacy and safety of Niaoduqing granules in the treatment of diabetic kidney disease: a systematic review and meta-analysis

**DOI:** 10.3389/fphar.2023.1180751

**Published:** 2023-07-05

**Authors:** Chaoqun Song, Zhiyue Zhu, Le Liu, Shilin Liu, Yuandong Li, Yang Xiao, Chunwei Wu, Zheng Nan

**Affiliations:** ^1^ Changchun University of Chinese Medicine, Changchun, China; ^2^ The Affiliated Hospital of Changchun University of Chinese Medicine, Changchun, China

**Keywords:** Niaoduqing granules, diabetic kidney disease, traditional Chinese medicine, systematic reviews, meta-analysis

## Abstract

**Background:** Diabetic nephropathy (DN) is the main cause of chronic kidney disease (CKD) and end-stage renal failure (ESRF), and the control of disease progression and adverse events during treatment needs to be improved.

**Objective:** This study aimed to systematically evaluate the clinical efficacy and safety of Niaoduqing granules (NDQG) in the treatment of diabetic kidney disease (DKD).

**Method:** Randomized controlled trials (RCTs) of NDQG for DKD from Chinese and English databases up to 31 August 2022 were included. The quality of the literature was assessed using the risk of bias tool of the Cochrane Handbook. At a 95% confidence interval (CI), relative risk (RR) and Cohen’s d were used for the categorical and continuous variables, respectively, and Stata 16.0 software was used for statistical analysis. A funnel plot and Egger’s tests were used to assess publication bias.

**Result:** A total of 4,006 patients were included in 52 RCTs, including 1,987 cases in the control group and 2,019 cases in the treatment group. Compared with conventional treatment (CT), combined NDQG therapy is more effective in improving clinical efficiency [RR = 1.23, 95% confidence interval (1.17, 1.29), *p* < 0.001, *I* ^*2*^ = 53.17%], kidney function (urinary albumin excretion rate [SMD = −0.90, 95% CI (−1.14, −0.66), *p* < 0.001, *I* ^*2*^ = 78.19%], 24hUTP levels [SMD = −0.81, 95% CI (−1.08, −0.55), *p* < 0.001, *I* ^*2*^ = 87.08%], blood urea nitrogen [SMD = −0.54, 95% CI (−0.69, −0.39), *p* < 0.01, *I* ^*2*^ = 77.01%], SCr [SMD = −0.68, 95% CI (−0.90, −0.45), *p* < 0.001, *I* ^*2*^ = 89.97%], CCr [SMD = 0.76, 95% CI (0.10,1.42), *p* = 0.02, *I* ^*2*^ = 95.97%], and Cys-C [SMD = −1.32, 95% CI (−2.25, −0.40), *p* = 0.01, *I* ^*2*^ = 93.44%]), the level of glucose metabolism (fasting blood glucose [SMD = −0.18, 95% CI (−0.38, 0.03), *p* = 0.10, *I* ^*2*^ = 71.18%] and HbA1c [SMD = −0.42, 95% CI (−0.86, −0.02), *p* = 0.06, *I* ^*2*^ = 81.64%]), the level of lipid metabolism (total cholesterol [SMD = −0.70, 95% CI (−1.01, −0.39), *p* < 0.001, *I* ^*2*^ = 86.74%] and triglyceride [SMD = −0.61, 95% CI (−0.87,−0.36), *p* < 0.001, *I* ^*2*^ = 80.64%]), inflammatory factors (Hs-CRP [SMD = −1.00, 95% CI (−1.54, −0.46), *p* < 0.001, *I* ^*2*^ = 86.81%], IL-18 [SMD = −1.25, 95% CI (−1.58, −0.92), *p* < 0.001, *I* ^*2*^ = 0], and TNF-α [SMD = −1.28, 95% CI (−1.64, −0.91), *p* < 0.001, *I* ^*2*^ = 75.73%]), and indicators of oxidative stress (malondialdehyde [SMD = −0.88, 95% CI (−1.22, −0.54), *p* < 0.001, *I* ^*2*^ = 66.01%] and advanced oxidation protein products [SMD = −0.92, 95% CI (−1.85, 0.00), *p* < 0.001, *I* ^*2*^ = 90.68%]). In terms of improving uric acid [SMD = −1.59, 95% CI (−3.45, 0.27), *p* = 0.09, *I* ^*2*^ = 94.67%], 2hPG [SMD = −0.04, 95% CI (−0.61, 0.53), *p* = 0.89, *I* ^*2*^ = 84.33%], HDL-C [SMD = 0.71, 95% CI (0.02, 1.40), *p* = 0.04, *I* ^*2*^ = 87.43%], Hb [SMD = 0.11, 95% CI (−0.10, 0.32), *p* = 0.32, *I* ^*2*^ = 0.00]), and superoxide dismutase [SMD = 1.32, 95% CI (0.44, 2.20), *p* < 0.001, *I* ^*2*^ = 93.48%], the effect is not obvious. Adjuvant treatment with NDQG did not increase the incidence of adverse reactions in the control group [SMD = 0.98, 95% CI (0.71, 1.34), *p* = 0.89, *I* ^*2*^ = 1.59%]. Obvious publication bias was detected by funnel plot and Egger’s test.

**Conclusion:** Our meta-analysis showed that adjuvant treatment with NDQG has more advantages than conventional treatment alone in the DKD treatment, which could improve clinical efficiency, kidney function, the level of glucose metabolism, the level of lipid metabolism, inflammatory factors, and oxidative stress indicators. At the same time, it also showed that NDQG are relatively safe. However, more high-quality studies are needed to provide more reliable evidence for clinical use.

**Systematic Review Registration**: https://www.crd.york.ac.uk/prospero/display_record.php?ID=CRD42022373726, identifier CRD42022373726.

## 1 Background

Diabetic kidney disease (DKD) is a chronic microvascular disease secondary to diabetes. DKD is the leading cause of chronic kidney disease (CKD) and end-stage renal failure (ESRF) worldwide (Collaboration, 2020), and DKD-associated CKD or ESRF mortality is higher in patients with CKD or ESRF than in non-DKD patients (Thomas, 2019). According to the data from the International Diabetes Federation in 2021, as of 2021, there are approximately 537 million adult diabetic patients aged 20 to 79, with a prevalence of 10.5%, although only 30%–40% of diabetic patients develop DKD, but it is the main cause of ESRF in most developed countries (Sun et al., 2022). The key features of DKD include persistent proteinuria, mesangial cell proliferation and stromal expansion, glomerulosclerosis, tubulointerstitial fibrosis, podocyte epithelial–mesenchymal transdifferentiation, and autophagy and apoptosis of podocytes (Hernandez et al., 2022). The treatment of DKD is mainly symptomatic treatment such as lowering blood pressure, controlling blood sugar, lowering lipids, improving circulation, and reducing proteinuria, but this does not prevent the progression of the disease, and it is accompanied by many adverse events (Gu et al., 2019). Recently, Traditional Chinese Medicine (TCM) has presented its unique advantages in the treatment of DKD, and previous studies have shown that TCM treatment can improve the clinical symptoms and quality of life of DKD patients, maintain the stability of the condition, and reduce adverse effects (Zhou et al., 2022).

NDQG have been approved by the China State Food and Drug Administration for the treatment of CRF as a compounded Chinese patent medicine and their productive process is under the national drug supervision and administration drug standards [WS3-229 (Z-033)-2000 (Z)], consisting of 16 herbs of TCM—rhubarb (Dahuang, *Rheum palmatum* L.), milkvetch root (Huangqi, *Astragalus mongholicus* Bunge [Fabaceae]), liquorice root (Gancao, *Glycyrrhiza glabra* L.), Largehead Atractylodes Rhizome (Baizhu, *Atractylodes macrocephala* Koidz.), Poria (Fuling, *Smilax glabra* Roxb.), Radix Polygoni Multiflori (Heshouwu, *Reynoutria multiflora* (Thunb.) Moldenke), Sichuan lovage rhizome (Chuanxiong, *Conioselinum anthriscoides* ‘Chuanxiong’), Chrysanthemum (Juhua, *Chrysanthemum indicum* L.), Salviae Miltiorrhizae (Danshen, *Salvia miltiorrhiza* Bunge), *Pinellia* tuber (Banxia, *Pinellia ternata* (Thunb.) Makino), Pilose Asiabell root (Dang shen, *Codonopsis pilosula* (Franch.) Nannf.), white mulberry root-bark (Sangbaipi, *Morus alba* L.), *Sophora japonica* (Kushen, *Sophora flavescens* Aiton), plantain (Cheqiancao, Plantago asiatica L.), white peony root (Baishao, Paeonia lactiflora Pall.), and Chinese thorowax root (Chaihu, *Bupleurum falcatum* L.)—with the effects of nourishing the kidney and filling essence, strengthening the spleen and dampness, reducing turbidity, activating blood, and removing stasis (Lin and Xu, 2017). In Traditional Chinese Medicine Systems Pharmacology Database and Analysis Platform (http://tcmspw.com/tcmsp.php), the screening criteria was set to DL ≥ 0.18, OB ≥ 30%, Caco-2 ≥ −0.4, and HL ≥ 4 (Zheng et al., 2016) and the drug ingredients were inquired, the composition of which is shown in Supplementary Material S1. As a TCM compound widely used in the treatment of chronic renal failure (CRF), azotemia phase, and early uremia, NDQG has a certain effect on nephrotic syndrome, and no obvious adverse reactions to the heart, liver, intestine, and other organs have been found after long-term medication (Lu et al., 2017), and it shows good efficacy in the prevention and treatment of CKD (10).

There are many clinical studies on the treatment of DKD with NDQG, but there is a lack of evidence-based medical support. This study aimed to investigate the efficacy and safety of NDQG through evidence-based medical approaches.

## 2 Data and methods

### 2.1 Registration

This systematic review and meta-analysis is the reporting item of choice for systematic reviews and meta-analyses (PRISMA) (Moher et al., 2009) conducted and reported under the guidance of the Cochrane Manual Systematic Review of Interventions Version 6.3 (2022 update). The PRISMA 2020 checklist is provided in Supplementary Material S2. Prior to the start of the 2020 statement, this study was registered in the International Prospective Register of Systematic Reviews (PROSPERO) (CRD42022373726). Data were derived from published clinical studies.

### 2.2 Database and search strategy

The search period was from the establishment of the library to 31 August 2022. The three English electronic databases of PubMed, Embase, and the Cochrane Library and the three Chinese electronic databases of CNKI (China National Knowledge Infrastructure), Wanfang Data, and VIP (China Science and Technology Journal Database) were comprehensively searched for RCTs on the treatment of DKD by NDQG, and the search was conducted by combining subject words and free words. The specific search strategies are shown in Table 1.

**TABLE 1:** Specific search strategies.

Number	Keywords of the research
#1	Diabetic nephropathy [mh] OR Diabetic nephropathies [mh]
#2	Diabetic nephropathy*[tiab] OR Diabetic kidney disease [tiab] OR Diabetic kidney lesion [tiab] OR Diabetic kidney damage [tiab] OR Diabetic renal disease [tiab] OR Diabetic renal lesion [tiab] OR Diabetic renal damage [tiab] OR renal dysfunction [tiab] OR DKD [tiab] OR CKD [tiab]
#3	#1 OR #2
#4	Niaoduqing [tiab] OR Niao Du Qing [tiab] OR NDQG [tiab]
#5	Randomized controlled trial [pt] OR Controlled clinical trial [pt]
#6	Randomized controlled trial [tiab] OR Controlled clinical trial [tiab] OR Randomized* [tiab] OR Randomly* [tiab] Random allocation [tiab] OR Trial [tiab] OR CCT [tiab] OR RCT [tiab]
#7	#5 OR #6
#8	#3 AND #4 AND #7

Note: mh, MeSH; tiab, title/abstract.

### 2.3 Inclusion criteria

#### 2.3.1 Type of study

RCTs of all original studies, irrespective of source or country, published in English or Chinese language only.

#### 2.3.2 Participants

Adult (at least 18 years old) patients diagnosed with DKD, regardless of age, gender, and course of the disease.

#### 2.3.3 Interventions

Placebo or usual treatment in the control group; the treatment group added NDQG to oral treatment on the basis of the same CT as the control group, or the treatment group added NDQG combined with CT. CT includes symptomatic treatment, diabetes health education, diet control, exercise intervention, daily monitoring of blood sugar changes, oral hypoglycemic drugs, insulin, and other classic Western medicine treatment measures.

#### 2.3.4 Outcome indicators

In order to comprehensively evaluate the efficacy and safety of NDQG for DKD, the clinical efficiency, kidney function, level of glucose metabolism, level of lipid metabolism, Hb, inflammatory factors, oxidative stress indicators, and adverse events were analyzed.

1) Primary outcome: clinical effective rate, urinary albumin excretion rate (UAER), and 24-h urine protein quantification (24UTP).
2) Secondary outcomes: blood urea nitrogen (BUN), serum creatinine concentration (SCr), creatinine clearance rate (CCr), cystatin-c (Cys-C), uric acid (UA), fasting blood glucose (FBG), 2-h postprandial blood glucose (2hPG), glycosylated hemoglobin (HbA1c), total cholesterol (TC), triglyceride (TG), high-density lipid-cholesterol (HDL-C), hemoglobin (Hb), high-sensitivity C-reactive protein (Hs-CRP), interleukin-18 (IL-18), tumor necrosis factor-α (TNF-α), malondialdehyde (MDA), superoxide dismutase (SOD), and advanced oxidation protein products (AOPP).

If a study reported multiple time points, the results with the longest time point were included in the analysis. If multiple stages were included in a study report, the first stage was analyzed.

3) Safety outcome: Any adverse events that occurred during the study should be recorded, such as the incidence of hypoglycemia, the rate of adverse events, the rate of serious adverse events, and the incidence of gastrointestinal adverse reactions.

### 2.4 Exclusion criteria

#### 2.4.1 Types of case reports

1) Non-RCT research studies, such as conference papers, animal studies, mechanism studies, clinical experience, data analysis, systematic reviews, guideline studies, retrospective studies, and data analysis and reviews.
2) If there were duplicates in the data, only the one with detailed data was selected.
3) Papers that could not be searched and authors who could not be contacted were excluded.
4) Literature from research in other fields, such as nursing, was excluded.

#### 2.4.2 Types of participants

1) Patients on dialysis were excluded.
2) Patients with other complications clearly written in the text were excluded.
3) Patients with unclear diagnostic criteria were excluded.

#### 2.4.3 Interventions

Interventions included TCM methods other than oral NDQG, such as enemas, acupuncture, and other TCM medicines.

#### 2.4.4 Outcome measures

There are obvious data errors or incomplete data and lack of required indicators.

### 2.5 Study selection and data extraction

Import the search results into the EndNoteX9 software in the form of a bibliography to establish a database. YDL (Yuandong Li) and YX (Yang Xiao) independently screened the literature, extracted the data, and cross-checked according to the inclusion criteria and exclusion criteria, and if there was a discrepancy, ZYZ (Zhiyue Zhu) was consulted to assist in judgment, and the authors of the literature lacking the data were contacted by email to obtain the literature as much as possible. First, software was used to delete duplicate documents, and then they were manually checked again to delete duplicate documents. Second, preliminary screening was carried out by reading the titles and abstracts of the literature, and literature that did not meet the criteria was excluded. Literature that still did not meet the criteria was excluded by reading the full text. If there were differences, they were determined after discussion or consultation with ZYZ. YDL and YX independently extracted data from the included studies and cross-checked them based on pre-designed data extraction tables. The data extraction content mainly included the following: (Collaboration, 2020) the basic information of the included research, the research title, first author, and year of publication; (Thomas, 2019) the baseline characteristics of the study subjects, including the sample size of each group, the age, gender, and disease status of the patients; (Sun et al., 2022) specific details of the intervention; (Hernandez et al., 2022) key elements of risk of bias assessment; and (Gu et al., 2019) outcomes of interest and adverse events.

### 2.6 Risk of bias assessment

CQS (Chaoqun Song) and SLL (Shilin Liu) assessed the risk of bias of the included studies according to the Cochrane risk of bias assessment tool for RCTs. The tool assessed seven important sources of bias: random sequence generation, allocation concealment, blinding of participants and personnel, blinding of outcome assessment, incomplete outcome data, selective reporting, and other biases. Risk of bias was assessed for each included study from these seven domains. By assessing the completeness of the study and the correctness of the methodological implementation, each aspect was assessed as “high risk,” “low risk, or “unclear risk.” The two researchers operated independently and examined each other’s results. Any disagreement on the evaluation of results was solved through discussion between LL (Le Liu) and CWW (Chunwei Wu) to make the final decision.

### 2.7 Statistical analysis

The meta-analysis was conducted using Stata 16.0 software, which is provided by the Cochrane Collaboration Network. Relative risk (RR) and its 95% Confidence interval (CI) were used as the combined effect size for the counted data. The mean difference (MD) and its 95% CI were used as the combined effect size for the measured data. *I* ^2^ was used for heterogeneity between results. Meta-analyses were performed using a fixed-effect model when there was statistical homogeneity between results (*p* > 0.1, *I* ^2^ < 50%), and sources of heterogeneity were analyzed if there was statistical heterogeneity between results (*p* < 0.1, *I* ^2^ > 50%). If there was statistical heterogeneity between groups, there was no clinical heterogeneity or difference. A random-effects model was used for meta-analysis. If there was too much heterogeneity between results, descriptive analysis was performed. Funnel plots were used to assess publication bias in results involving more than 10 studies. Subgroup analysis based on prespecified assumptions explored sources of heterogeneity if necessary.

## 3 Results

### 3.1 Literature screening process and results

**FIGURE 1:**
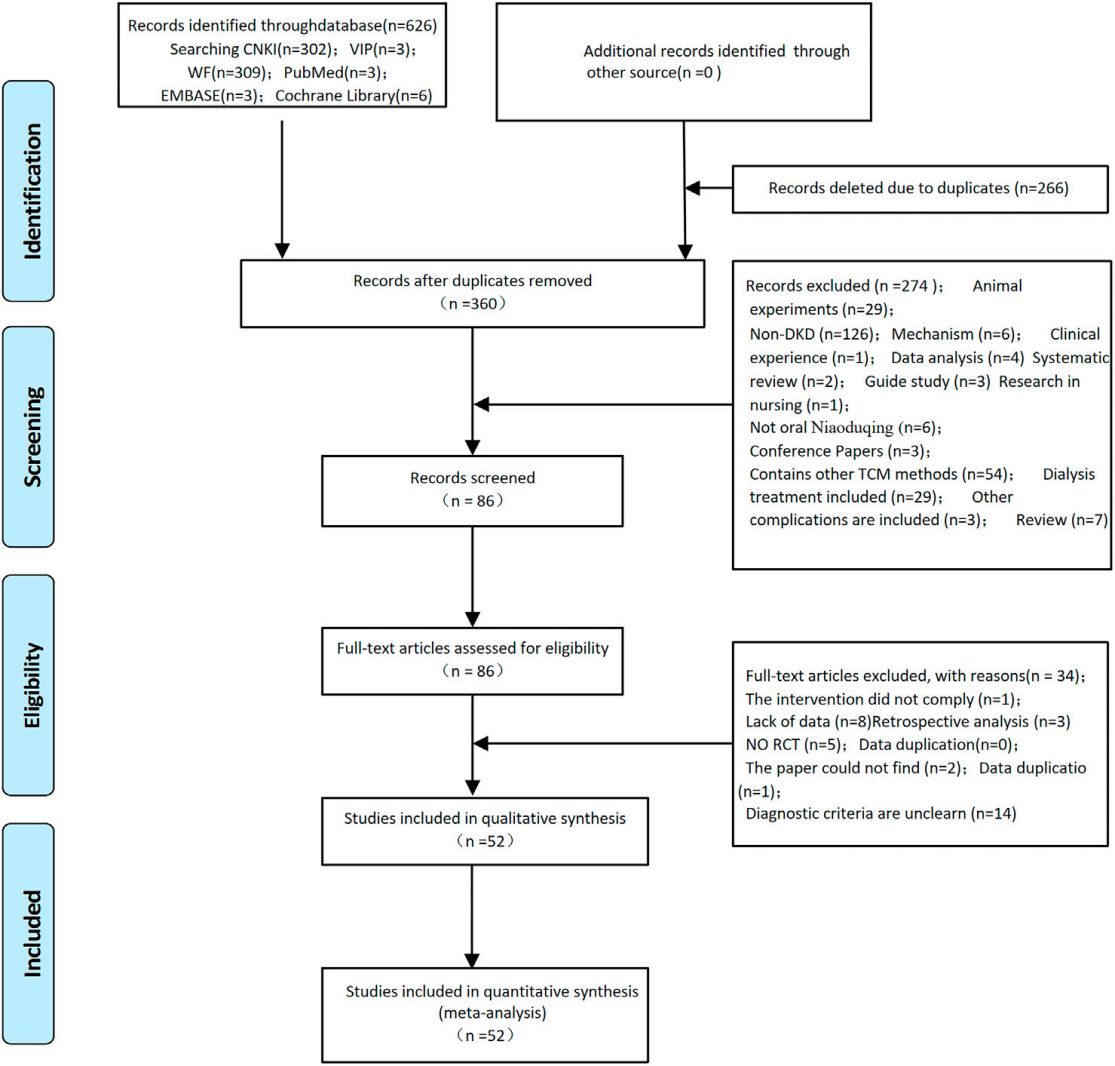
Flowchart of study selection and identification.

Through the search of six major Chinese and English databases, 626 relevant articles were obtained. Of these, 266 were excluded due to duplication. After reading the article titles and abstracts, 274 were excluded. By reading the full text of the remaining 86 papers, a total of 52 papers (Cao and Liu, 2005; Lv et al., 2008; Zhao et al., 2008; Wang et al., 2009; Wu et al., 2009; Xie and Feng, 2009; Yang, 2009; Zhao and Chen, 2009; Bai and Shi, 2010; Cai et al., 2010; Cai and Wang, 2010; Jia et al., 2010; Yan and Liu, 2010; Zhang et al., 2010; Chen, 2011; Liang, 2011; Wang and Zhang, 2011; Xiao et al., 2011; Zhang et al., 2011; Hu, 2012; Liu et al., 2012; Wang et al., 2012; Fan, 2013; Gong, 2013; Wang et al., 2013; Yao and Ren, 2013; Yu, 2013; Zhang, 2013; Shi et al., 2014; Hao, 2015; He and Ding, 2015; Huang, 2015; Li, 2015; Wan, 2015; Li et al., 2016; Wang, 2016; Zhang et al., 2016; Li et al., 2017; Luo and Li, 2018; Wei and Ruan, 2018; Ding and Zhang, 2019; Liu and Yang, 2020; Chen et al., 2021; Fang, 2021; He, 2021; Kou, 2021; Lan et al., 2021; Li et al., 2021; Liang et al., 2021; Wu, 2021; Xu, 2021; Li, 2022) were finally included for quantitative analysis. The study selection and identification are shown in Figure 1.

### 3.2 Basic characteristics of the included studies

The total of 52 RCTs included in this study, all of which are Chinese literature, were dated between 2005 and 2022, and a total of 4,006 patients were included, including 1,987 in the control group and 2,019 in the treatment group. The WHO, ADA, Morganson, or Chinese guidelines were referred to for diagnostic criteria. The baseline characteristics for inclusion in this study are shown in Table 2.

**TABLE 2:** Baseline characteristics for inclusion.

Study (author/year)	Sample size (T/C)	Gender (M/F; T/C	Average age T/C)	Duration of treatment	Intervention group	Control group (regimen)	Main outcome
Wang et al. (2012)	61/61	72/50	63.5 ± 4.2	2 months	NDQG + CT	CT + Medicine Charcoal Table	①③④⑤⑥⑮㉒
Zhao et al. (2008)	68/68	73/63	48.2	2 months	NDQG + CT	CT	①②⑨⑪⑫⑬⑭
He and Ding. (2015)	24/24	13/11	47.15 ± 3.53	2 months	NDQG + CT	CT	①③④⑤⑨⑩⑫⑬
		14/10	46.75 ± 3.26				
Li. (2022)	51/51	32/19	56.32 ± 6.02	14 days	NDQG + CT + Baxter Pharmaceutical Solutions	CT + Baxter Pharmaceutical Solutions	①③④⑤㉒
		33/18	56.38 ± 6.07				
Wei and Ruan, (2018)	45/45	28/17	64.81 ± 7.97	3 months	NDQG + CT + olmesartan	CT + olmesartan	①④⑥⑨⑪⑦⑮㉒
		26/19	65.31 ± 8.37				
Li et al. (2021)	40/40	24/16	63.24 ± 11.36	3 months	NDQG + CT + irbesartan	CT + irbesartan	①④⑤⑥⑯⑰⑱⑲⑳㉑㉒
		27/13	64.55 ± 11.24				
Cai and Wang (2010)	40/40	25/15	56 ± 8	3 months	NDQG + CT + calcium dobesilate capsules	CT + calcium dobesilate capsules	②④⑤⑨⑫⑬
		28/12	53 ± 6				
Lv et al. (2008)	34/37	21/14	45.2 ± 10.6	3 months	NDQG + Vitamin E capsules + CT	CT	①③④⑤⑨㉒
		19/11	42.5 ± 13.1				
He (2021)	44/43	23/21	62.2 ± 8.5	3 months	NDQG + CT + valsartan	CT + valsartan	①②③④⑤⑰⑱㉒
		24/19	61.5 ± 8.3				
Jia et al. (2010)	25/25	30/20	54	12 weeks	NDQG + CT	CT	③④⑤⑨⑫⑬⑲
Zhao and Chen (2009)	34/34	Unclear	Unclear	3 months	NDQG + CT	CT	①④⑤⑥
Liang (2011)	27/23	26/24	59.9 ± 8.3	90 days	NDQG + CT	CT	①③④⑤⑨
Cao and Liu (2005)	32/30	18/14	66.7	2 months	NDQG + CT	CT	①⑤⑥
		19/11	64.91				
Huang (2015)	33/33	19/14	62.6 ± 6.5	1 month	NDQG + CT	CT + Medicine Charcoal Table orally	①③④⑤⑥
		18/15	63.4 ± 6.9				
Yang (2009)	40/40	42/38	52.8	6 months	NDQG + CT	CT	②⑥⑫⑬
Li et al. (2016)	34/34	19/15	57.6 ± 6.5	3 months	NDQG + CT + ACEI/ARB	CT + ACEI/ARB	③④⑤⑪㉒
		17/17	64.3 ± 5.9				
Wang and Zhang, (2011)	40/40	19/21	52.7 ± 8.2	4 weeks	NDQG + CT	CT	①④⑤⑫⑬
		28/12	49.3 ± 7.6				
Cai et al. (2010)	33/33	18/15	52 ± 5	3 months	NDQG + CT	CT	②④⑤⑨⑫⑬
		19/14					
Xu (2021)	38/27	25/13	53.09 ± 5.69	8 weeks	NDQG + CT + piperazine ferulate	CT + piperazine ferulate	①③④⑤⑧
		17/10	53.47 ± 5.90				
Liu and Yang (2020)	78/78	Unclear	67.20 ± 9.42 65.19 ± 7.31	6 months	NDQG + CT + insulin detemir	CT + insulin detemir	①②④⑤⑨⑩⑱⑲⑳㉑㉒
Chen (2011)	24/24	28/20	Unclear	3 weeks	NDQG + CT + alprostadil injection	CT + alprostadil injection	②
Wang et al. (2009)	32/32	20/12	57.45 ± 9.82	6 months	NDQG + CT + telmisartan	CT + telmisartan	②④⑤⑨⑫⑬⑭
		18/14	58.12 ± 8. 28				
Zhang et al. (2010)	33/33	38/28	Unclear	12 weeks	NDQG + CT	CT	①③④⑤⑥⑨⑩⑫⑬
Fang (2021)	60/60	31/29	Unclear	Not mentioned	NDQG + CT + benazepril hydrochloride tablets	CT + benazepril hydrochloride tablets	①④⑤
		28/32					
Chen et al. (2021)	41/41	26/15	51.40 ± 1.38	3 months	NDQG + CT + insulin degludec and insulin aspart	CT + Insulin degludec and insulin aspart	①③④⑤⑥⑯⑱⑲⑳㉒
		25/16	51.38 ± 2.46				
Ding and Zhang (2019)	30/30	17/13	60.9 ± 10.3	4 weeks	NDQG + CT + Alisartan Cilexetil	CT + Allisartan Isoproxil Tables	①③④⑤㉒
		16/14					
Kou (2021)	46/46	29/17	96 ± 5.78	3 months	NDQG + CT + olmesartan	CT + olmesartan	①③④⑤㉒
		27/19	49.49 ± 5.81				
Gong (2013)	30/27	17/14	59.4 ± 7.5	3 months	NDQG + CT + irbesartan	CT + irbesartan	②④⑤⑨⑫⑬⑯
		15/16	60.0 ± 7.3				
Lan et al. (2021)	41/41	28/13	60.15 ± 4.02	3 months	NDQG + CT + losartan potassium	CT + losartan potassium	①④⑤⑲⑳㉒
		26/15	60.08 ± 4.17				
Yan and Liu (2010)	33/33	19/14	53 ± 6	3 months	NDQG + vitamin E capsules + CT	CT	①②④⑤⑨⑫⑬
		18/15	52 ± 5				
Hu (2012)	18/20	22/16	64	2 months	NDQG + CT + valsartan	CT + valsartan	③⑤
Zhang (2013)	30/31	16/14	55.12 ± 4.7	4 weeks	NDQG + CT	CT + aldehyde oxygen starch capsules	①③④⑤
		15/16	54.53 ± 3.94				
Hao (2015)	24/24	12/12	42.8 ± 10.2	1 month	NDQG + CT + ACEI/ARB	CT + ACEI/ARB	①②③④⑤
		14/10	45.2 ± 10.8				
Wan (2015)	30/28	Unclear	Unclear	8 weeks	NDQG + alprostadil injection + CT	CT	①②④⑤⑧㉒
Li (2015)	45/45	44/46	52.2 ± 6.3	60 days	NDQG + CT	CT	③④⑤
Liu et al. (2012)	30/30	18/12	56.35 ± 9.82	6 months	NDQG + CT + irbesartan	CT + irbesartan	④⑤
		20/10	56.45 ± 9.86				
Liang et al. (2021)	49/48	27/22	50.05 ± 12.99	4 weeks	NDQG + CT + compound α-ketoacid tablets	CT + compound α-ketoacid tablets	①③④⑤⑱㉒
		26/22	50.01 ± 13.01				
Zhang et al. (2011)	40/40	49/31	54. 6 ± 7. 5	60 days	NDQG + CT	CT	④⑤⑯
Wang et al. (2013)	68/65	69/64	50.42	24 weeks	NDQG + CT + ACEI/ARB	CT + ACEI/ARB	①⑥⑨⑪⑦⑫⑬⑮㉒
Wu (2021)	41/41	27/14	87 ± 3.69	1 month	NDQG + CT + valsartan	CT + valsartan	②④⑤⑯⑱㉒
		26/25					
Zhang et al. (2016)	40/38	26/14	65.14 ± 11.26	1 month	NDQG + CT + valsartan	CT + valsartan	①③④⑤
		27/11	64.83 ± 10.74				
Li (2016)	60/60	Unclear	Unclear	4 weeks	NDQG + captopril + CT	CT	①③④⑤㉒
Yao and Ren (2013)	30/30	16/14	52.9 ± 7.9	2 months	NDQG + CT + alpha lipoic acid injection	CT + Alpha lipoic acid injection	①②
		17/13	53.5 ± 7.8				
Luo and Li (2018)	50/50	62/38	53.1	6 months	NDQG + CT	CT	⑤⑥⑫⑬
Xie and Feng (2009)	25/23	18/7	45.5 ± 8.8	3 months	NDQG + CT	CT	③④⑤⑨⑪⑭
		18/5	43.3 ± 10.2				
Wu et al. (2009)	38/38	43/33	Unclear	12 weeks	NDQG + CT	CT	①③④⑤⑥⑨⑩⑫⑬
Fan (2013)	30/30	16/14	63.2 ± 2.2	3 months	NDQG + CT + benazepril hydrochloride tablets	CT + benazepril hydrochloride tablets	①③④⑤⑫⑬
		15/15	62.8 ± 2.1				
Bai and Shi (2010)	30/30	19/11	Unclear	3 months	NDQG + CT + ACEI/ARB	CT + ACEI/ARB	③④⑤⑫⑬㉒
		17/13					
Xiao et al. (2011)	40/40	21/19	Unclear	90 days	NDQG + CT	CT	⑥⑤⑦㉒
		22/18					
Shi et al. (2014)	29/28	17/12	51.3 ± 9.2	12 weeks	NDQG + CT + perindopril	CT + perindopril	②④⑤⑨⑦⑫⑬⑭
		17/11	50.1 ± 8.7				
Yu (2013)	36/40	Unclear	Unclear	14 days	NDQG + alprostadil injection	CT	③④⑤⑫⑬
Wang (2016)	45/45	44/46	52.2 ± 6.3	60 days	NDQG + CT	CT	②④⑤⑯

① Clinically effective; ② UAER; ③ 24hUTP; ④ BUN; ⑤ SCR; ⑥ CCr; ⑦ Cys-C; ⑧ UA; ⑨ FBG; ⑩ 2hPG; ⑪ HbA1c; ⑫ TC; ⑬ TG; ⑭ HDL-C; ⑮ Hb; ⑯ Hs-CRP; ⑰ IL-18; ⑱ TNF-α; ⑲ MOD; ⑳ SOD; ㉑ AOPP; ㉒ adverse reactions.

Note: CT, conventional treatment; NDQG, Niaoduqing granules.

### 3.3 Risk of bias assessment

A total of 52 RCTs were included in this systematic review, of which 15 studies (Cai et al., 2010; Cai and Wang, 2010; Yan and Liu, 2010; Wang et al., 2013; He and Ding, 2015; Li, 2015; Wang, 2016; Zhang et al., 2016; Wei and Ruan, 2018; He, 2021; Kou, 2021; Lan et al., 2021; Liang et al., 2021; Wu, 2021; Li, 2022) used the random number table method, one study (Liu and Yang, 2020) used the random lottery method, and the remaining 36 items (Cao and Liu, 2005; Lv et al., 2008; Zhao et al., 2008; Wang et al., 2009; Wu et al., 2009; Xie and Feng, 2009; Yang, 2009; Zhao and Chen, 2009; Bai and Shi, 2010; Jia et al., 2010; Zhang et al., 2010; Chen, 2011; Liang, 2011; Wang and Zhang, 2011; Xiao et al., 2011; Zhang et al., 2011; Hu, 2012; Liu et al., 2012; Wang et al., 2012; Fan, 2013; Gong, 2013; Yao and Ren, 2013; Yu, 2013; Zhang, 2013; Shi et al., 2014; Hao, 2015; Huang, 2015; Wan, 2015; Li et al., 2016; Li et al., 2017; Luo and Li, 2018; Ding and Zhang, 2019; Chen et al., 2021; Fang, 2021; Li et al., 2021; Xu, 2021) mentioned randomization but did not describe the specific random method, and the aforementioned literature was not sufficiently blinded. In one reference (Lv et al., 2008), the results and description of the UAER did not match, and this set of data was excluded; the data of TC and TG in one paper (Jia et al., 2010) were reversed, and the results were changed back after verifying the authors; the verified results and the unit errors for CCr in one study (Liang, 2011) and 24hUTP in two studies (Chen et al., 2021; Kou, 2021) were not excluded; the 24hUTP unit was wrong in one study (Wang and Zhang, 2011), and we cannot identify or correct it, so it was excluded; the UAER unit was wrong in one study (Liu et al., 2012), and the data were excluded. Three RCTs (Lv et al., 2008; Gong, 2013; Wan, 2015) had participants of exfoliation, which were not included, and exfoliation cases were excluded. There were multiple sets of data for the same indicator in three papers (Yang, 2009; Yao and Ren, 2013; Luo and Li, 2018), and the group of data with a longer observation period was selected. Overall, the quality of the included literature was not high.

## 4 Outcomes of meta-analysis

### 4.1 Primary outcome

#### 4.1.1 Clinical effective rate

**FIGURE 2:**
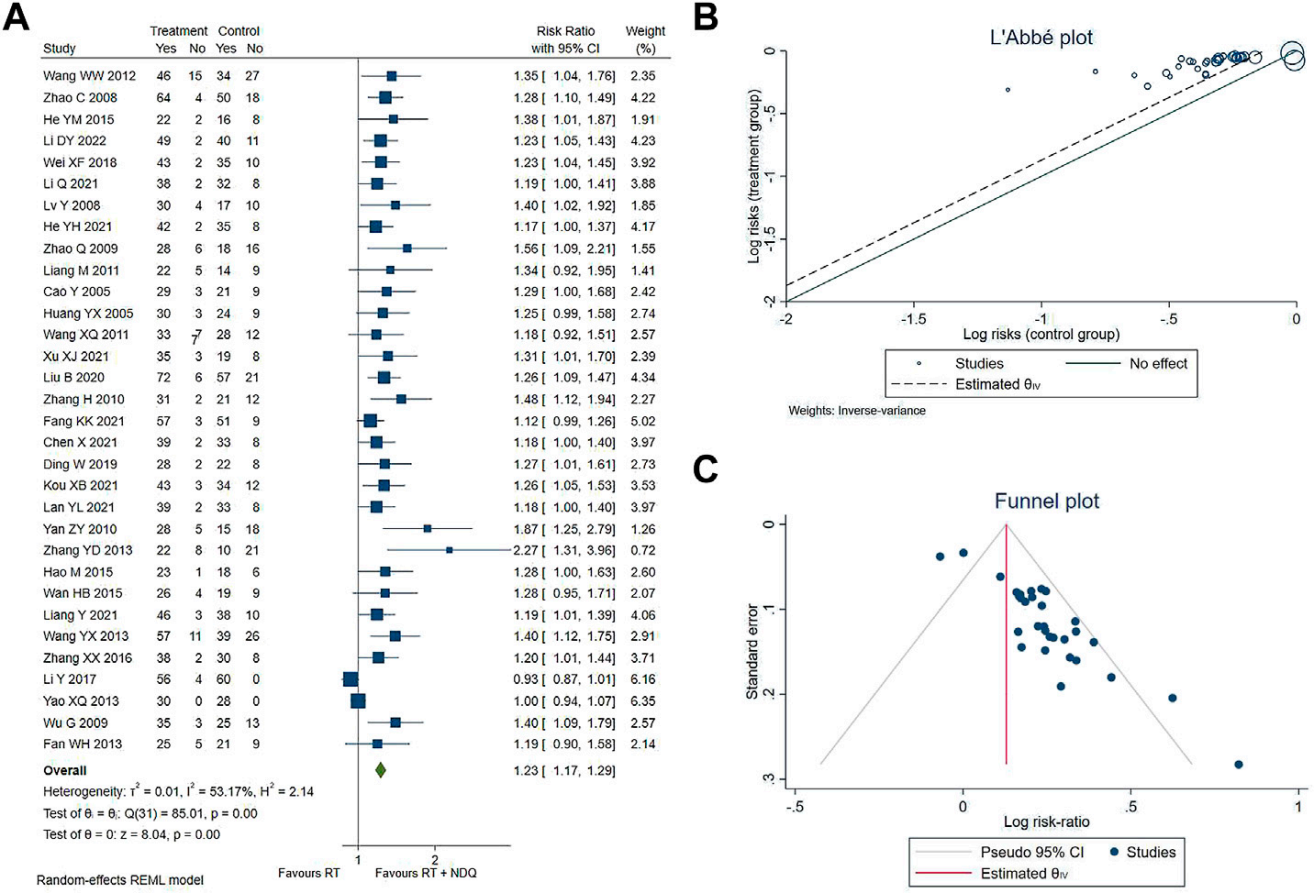
**(A)** Clinically effective. **(B)** L'Abbe plot. **(C)** Funnel plot.

The clinical effective rate were reported in 32 studies (Cao and Liu, 2005; Lv et al., 2008; Zhao et al., 2008; Wu et al., 2009; Zhao and Chen, 2009; Yan and Liu, 2010; Zhang et al., 2010; Liang, 2011; Wang and Zhang, 2011; Wang et al., 2012; Fan, 2013; Wang et al., 2013; Yao and Ren, 2013; Zhang, 2013; Hao, 2015; He and Ding, 2015; Huang, 2015; Wan, 2015; Zhang et al., 2016; Li et al., 2017; Wei and Ruan, 2018; Ding and Zhang, 2019; Liu and Yang, 2020; Chen et al., 2021; Fang, 2021; He, 2021; Kou, 2021; Lan et al., 2021; Li et al., 2021; Liang et al., 2021; Xu, 2021; Li, 2022) involving 2,630 patients, including 1,332 in the treatment group and 1,298 in the control group. The results showed that the clinical effective rate of the treatment of the experimental group was better than that of the control group [RR = 1.23, 95% CI (1.17, 1.29), *p* < 0.001, *I* ^2^ = 53.17%], which was statistically significant, as shown in Figure 2A. In the included literature, heterogeneity analysis showed that heterogeneity was due to the difference in the specific conventional treatment modality of the different DKD stages of the patients. L'Abbe diagrams and funnel diagrams are shown in Figures 2B, C.

#### 4.1.2 UAER

**FIGURE 3:**
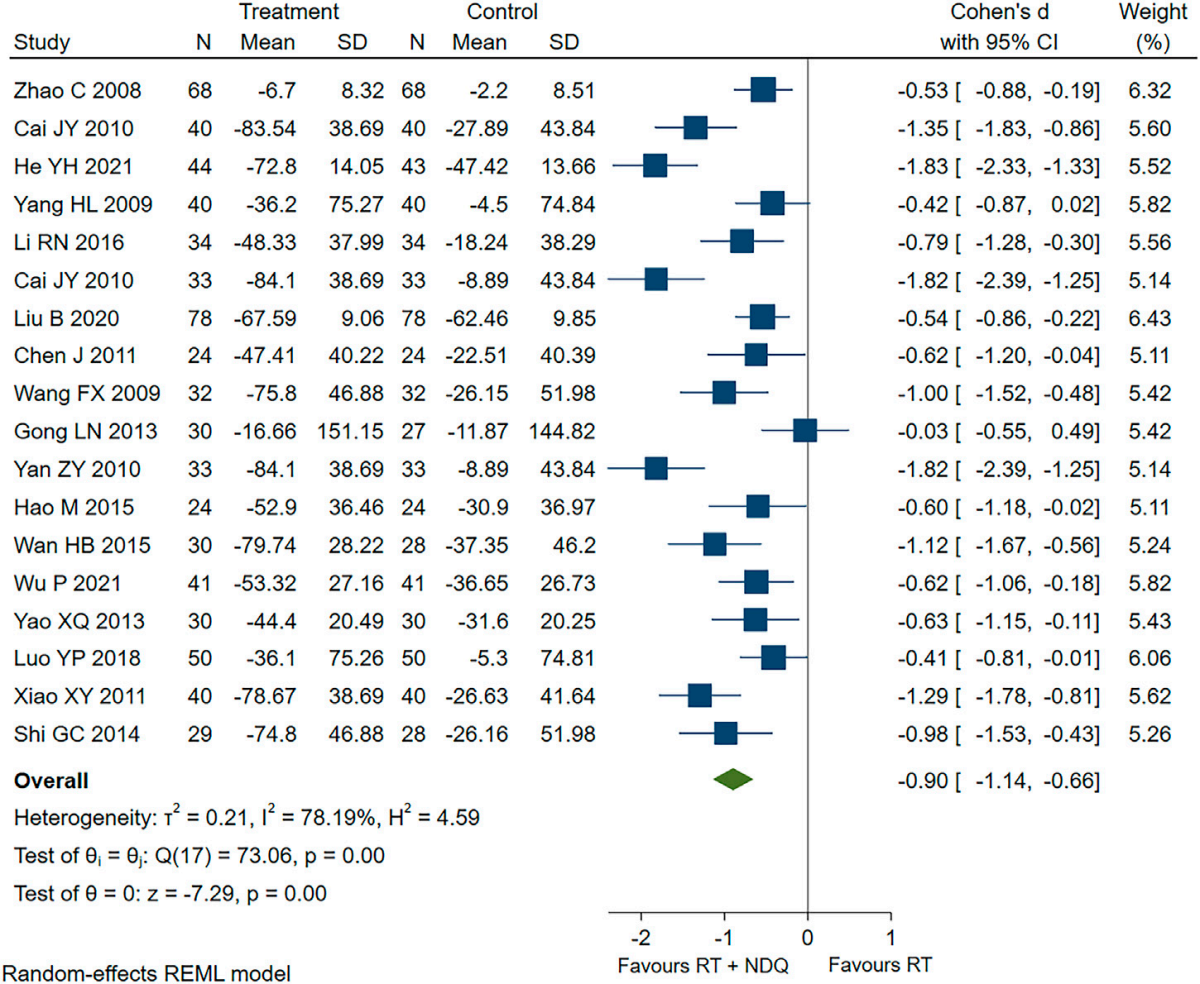
UAER forest chart.

The UAER levels were reported in 18 studies (Zhao et al., 2008; Wang et al., 2009; Yang, 2009; Cai et al., 2010; Cai and Wang, 2010; Yan and Liu, 2010; Chen, 2011; Xiao et al., 2011; Gong, 2013; Yao and Ren, 2013; Shi et al., 2014; Hao, 2015; Wan, 2015; Li et al., 2016; Luo and Li, 2018; Liu and Yang, 2020; He, 2021; Wu, 2021), including 700 patients in the treatment group and 693 patients in the control group. The results showed that the treatment group significantly outperformed the control group in reducing UAER levels [SMD = −0.90, 95% CI (−1.14, −0.66), *p* < 0.001, *I* ^2^ = 78.19%], as shown in Figure 3.

#### 4.1.3 24hUTP

**FIGURE 4:**
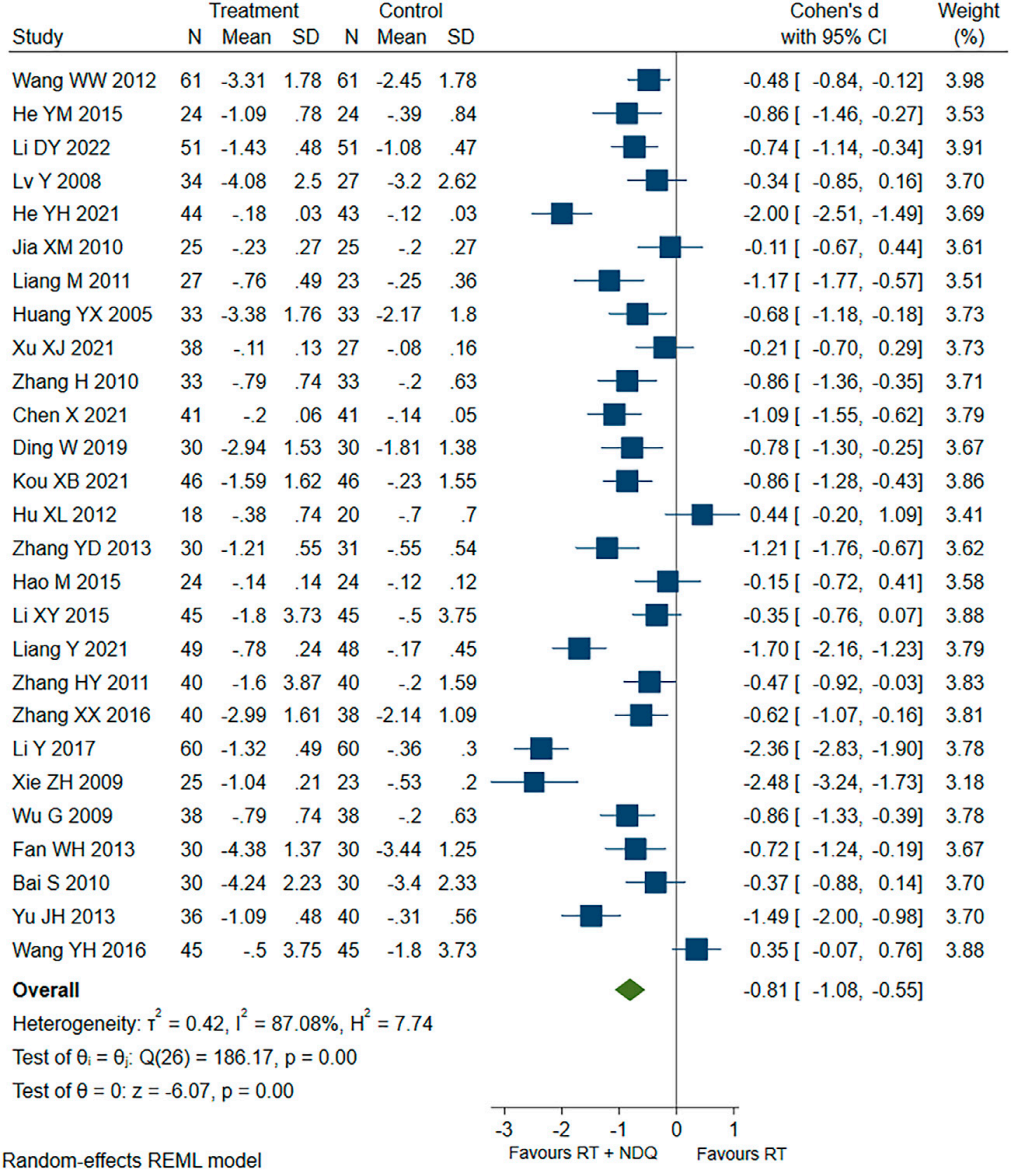
24hUTP forest chart.

The 24hUTP levels were reported in 27 studies (Lv et al., 2008; Wu et al., 2009; Xie and Feng, 2009; Bai and Shi, 2010; Jia et al., 2010; Zhang et al., 2010; Liang, 2011; Zhang et al., 2011; Hu, 2012; Wang et al., 2012; Fan, 2013; Yu, 2013; Zhang, 2013; Hao, 2015; He and Ding, 2015; Huang, 2015; Li, 2015; Wang, 2016; Zhang et al., 2016; Li et al., 2017; Ding and Zhang, 2019; Chen et al., 2021; He, 2021; Kou, 2021; Liang et al., 2021; Xu, 2021; Li, 2022), including 997 patients in the treatment group and 976 patients in the control group. The results showed that the treatment group was significantly better than the control group in reducing 24hUTP levels [SMD = −0.81, 95% CI (−1.08, −0.55), *p* < 0.001, *I* ^2^ = 87.08%], and the forest chart is shown in Figure 4.

### 4.2 Secondary outcomes and safety outcomes

We studied 18 secondary outcomes from six aspects: kidney function, the level of glucose metabolism, the level of lipid metabolism, hemoglobin, inflammatory factors, and oxidative stress. Furthermore, safety outcomes were studied from adverse events, the specific results of which are shown in Table 3; forest charts are shown in Supplementary Materials S3–S21.

**TABLE 3:** Secondary outcomes and safety outcomes.

Outcome measure	Included in the literature	Quantity	N (T/C0)	Testing for heterogeneity		Meta-analysis	
				*I* ^*2*^ */*%	*P*	*SMD*	*95% CI*
BUN	Bai and Shi (2010); Cai et al. (2010); Cai and Wang. (2010), Fan (2013); Gong (2013); Hao (2015); He and Ding (2015); Ding and Zhang (2019); Chen et al. (2021); Fang (2021); He (2021), Jia et al. (2010); Liang (2011); Liu et al. (2012); Huang (2015); Li (2015); Li et al. (2016); Li et al. (2017); Liu and Yang (2020); Kou (2021); Lan et al. (2021); Li et al. (2021); Liang et al. (2021); Li (2022), Lv et al. (2008); Wang et al. (2009); Wang and Zhang (2011); Wang et al. (2012); Shi et al. (2014); Wan (2015); Wang (2016); Wu et al. (2009); Wei and Ruan (2018); Wu (2021), Xie and Feng (2009); Yan and Liu (2010); Xu (2021), Zhang et al. (2010); Zhang et al. (2011); Yu (2013); Zhang (2013); Zhang et al. (2016), Zhao and Chen (2009)	43	1,649/1,620	77.01	<0.001	−0.54	−0.69, −0.39
SCr	Cao and Liu (2005); Bai and Shi (2010); Cai et al. (2010); Cai and Wang. (2010); Jia et al. (2010); Liang (2011); Hu (2012); Liu et al. (2012); Fan (2013); Gong (2013); Hao (2015); He and Ding (2015); Huang (2015); Li (2015); Li et al. (2016); Li et al. (2017); Ding and Zhang (2019); Liu and Yang (2020); Chen et al. (2021); Fang (2021); He (2021); Kou (2021); Lan et al. (2021); Li et al. (2021); Liang et al. (2021); Li (2022); Lv et al. (2008); Wang et al. (2009); Wang and Zhang (2011); Wang et al. (2012); Shi et al. (2014); Wan (2015); Wang (2016); Wu et al. (2009); Xie and Feng (2009); Yan and Liu (2010); Xiao et al. (2011); Wu (2021); Xu (2021); Zhang et al. (2010); Zhang et al. (2011); Yu (2013); Zhang (2013); Zhang et al. (2016); Zhao and Chen (2009)	45	1,694/1,665	89.97	<0.001	−0.68	−0.90, −0.45
CCr	Cao and Liu (2005); Yang (2009); Zhao and Chen (2009); Zhang et al. (2010); Xiao et al. (2011); Wang et al. (2012); Wang et al. (2013); Huang (2015); Luo and Li (2018); Wei and Ruan (2018); Chen et al. (2021); Li et al. (2021)	12	515/510	95.97	= 0.02	0.76	0.10, 1.42
Cys-C	Xiao et al. (2011); Wang et al. (2013); Shi et al. (2014); Wei and Ruan (2018)	4	182/178	93.44	= 0.01	−1.3	−2.25, −0.40
UA	Wan, 2015; Xu (2021)	2	65/58	94.67	= 0.09	−1.59	−3.45, 0.27
FBG	Lv et al. (2008); Zhao et al. (2008); Wang et al. (2009); Wu et al. (2009); Xie and Feng (2009); Cai et al. (2010); Cai and Wang (2010); Jia et al. (2010); Yan and Liu (2010); Zhang et al. (2010); Liang (2011); Gong. (2013); Wang et al. (2013); Shi et al. (2014); He and Ding (2015); Wei and Ruan (2018); Liu and Yang (2020)	17	662/642	71.18	= 0.10	−0.18	−0.38, 0.03
2hPG	Wu et al. (2009); Zhang et al. (2010); He and Ding (2015); Liu and Yang (2020)	4	173/173	84.33	= 0.89	−0.04	−0.61, 0.53
HbA1c	Zhao et al. (2008); Xie and Feng (2009); Wang et al. (2013); He and Ding (2015); Li et al. (2016)	5	240/235	81.64	= 0.06	−0.42	−0.86, −0.02
TC	Zhao et al. (2008); Wang et al. (2009); Wu et al. (2009); Yang (2009); Bai and Shi (2010); Cai et al. (2010); Cai and Wang (2010); Jia et al. (2010); Yan and Liu (2010); Zhang et al. (2010); Wang and Zhang. (2011); Fan (2013); Gong. (2013); Wang et al. (2013); Yu (2013); Shi et al. (2014); He and Ding (2015); Luo and Li (2018)	18	679/676	86.74	<0.001	−0.70	−1.01, −0.39
TG	Zhao et al. (2008); Wang et al. (2009); Wu et al. (2009); Yang (2009); Bai and Shi (2010); Cai et al. (2010); Cai and Wang (2010); Jia et al. (2010); Yan and Liu (2010); Zhang et al. (2010); Wang and Zhang (2011); Fan (2013); Gong (2013); Wang et al. (2013); Yu (2013); Shi et al. (2014); He and Ding (2015); Luo and Li (2018)	18	679/676	80.64	<0.001	−0.61	−0.87, −0.36
HDL-C	Zhao et al. (2008); Wang et al. (2009); Xie and Feng (2009); Shi et al. (2014)	4	154/151	87.43	= 0.04	0.71	0.02, 1.40
Hb	Wang et al. (2012); Wang et al. (2013); Wei and Ruan (2018)	3	174/111	0.00	= 0.32	0.11	−0.10, 0.32
Hs-CRP	Zhang et al. (2011); Gong (2013); Wang (2016); Chen et al. (2021); Li et al. (2021); Wu (2021)	6	237/234	86.81	<0.001	−1.00	−1.54, −0.46
IL-18	He (2021); Li et al. (2021)	2	84/83	0.00	<0.001	−1.25	−1.58, −0.92
TNF-α	Liu and Yang (2020); Chen et al. (2021); He (2021); Li et al. (2021); Liang et al. (2021); Wu (2021)	6	293/291	75.73	<0.001	−1.28	−1.64, −0.91
MDA	Jia et al. (2010); Liu and Yang (2020); Chen et al. (2021); Lan et al. (2021); Li et al. (2021)	5	225/225	66.01	<0.001	−0.88	−1.22, −0.54
SOD	Liu and Yang (2020); Chen et al. (2021); Lan et al. (2021); Li et al. (2021)	4	200/200	93.48	<0.001	1.32	0.44, 2.20
AOPP	Liu and Yang (2020); Li et al. (2021)	2	118/118	90.68	<0.001	−0.92	−1.85, 0.00
Adverse effect	Lv et al. (2008); Bai and Shi (2010); Xiao et al. (2011); Wang et al. (2012); Wang et al. (2013); Wan (2015); Li et al. (2016); Li et al. (2017); Wei and Ruan (2018); Ding and Zhang (2019); Liu and Yang (2020); Chen et al. (2021); He (2021); Kou (2021); Lan et al. (2021); Li et al. (2021); Liang et al. (2021); Wu (2021); Li (2022)	19	863/849	1.59	= 0.89	0.98	0.71, 1.34

Note: *I^2^*/%, heterogeneity/%; P, *p*-value; *SMD*, the standardized mean difference; *95% CI*, 95% confidence interval (CI).

## 5 Discussion

### 5.1 Principal findings of this research

Clinical studies have shown that diabetes is more likely to cause glomerulosclerosis in affected patients leading to renal insufficiency, which leads to DKD (Li, 2016; Jing et al., 2017). Increased deposition of extracellular matrix proteins at the glomerular level, such as fibronectin and type IV (IV-C), leads to thylakoid dilation and thickening of the glomerular basement membrane, leading to progressive renal failure, which eventually develops into ESRF (Lan et al., 2010).

In this study, we analyzed the efficacy and safety of NDQG in the treatment of DKD for the first time and conducted detailed analyses of the indicators in terms of clinical effective rate, kidney function, the level of glucose metabolism, the level of lipid metabolism, Hb, inflammatory factors, oxidative stress, and adverse events. A total of 626 articles were identified, and 52 were included. The risk of bias assessment showed that the methodological quality of the included studies was low, mainly due to lack of detailed reporting of specific methods for randomization and allocation concealment and inadequate blinding.

This meta-analysis found that compared to CT alone, adjuvant with NDQG had significantly better clinical efficacy in the treatment of patients with DKD [RR = 1.23, 95% CI (1.17, 1.29), *p* < 0.001, *I* ^*2*^ = 53.17%]. Therefore, the clinical use of NDQG as an adjuvant treatment of DKD is a good choice.

NDQG are generally beneficial in improving kidney function. NDQG can significantly reduce UAER levels [SMD = −0.90, 95% CI (−1.14, −0.66), *p* < 0.001, *I* ^*2*^ = 78.19%], 24hUTP levels [SMD = −0.81, 95% CI (−1.08, −0.55), *p* < 0.001, *I* ^*2*^ = 87.08%], BUN [SMD = −0.54, 95% CI (−0.69, −0.39), *p* < 0.01, *I* ^*2*^ = 77.01%], SCr [SMD = −0.68, 95% CI (−0.90, −0.45), *p* < 0.001, *I* ^*2*^ = 89.97%], and Cys-C [SMD = −1.32, 95% CI (−2.25, −0.40), *p* = 0.01, *I* ^*2*^ = 93.44%] and elevate CCr [SMD = 0.76, 95% CI (0.10, 1.42), *p* = 0.02, *I* ^*2*^ = 95.97%], but the reduction of UA [SMD = −1.59, 95% CI (−3.45, 0.27), *p* = 0.09, *I* ^*2*^ = 94.67%] is not much different from CT. Therefore, we can think that NDQG improve kidney function by reducing UAER, 24hUTP, SCr, BUN, and Cys-C and increasing CCr but have little effect on UA. The level of estimated glomerular filtration rate (eGFR) is a common indicator used to evaluate whether the kidney function is normal (You et al., 2018). However, only one paper (Kou, 2021) observed this indicator, but it is not sufficient for pooled analysis, so eGFR is not discussed in this paper. Relevant studies have shown that NDQG can improve renal fibrosis by inhibiting TGF-β1 expression, reducing podocyte damage, inhibiting tubular epithelial cell transdifferentiation, improving the microinflammatory state, inhibiting oxidative stress, and reducing insulin resistance (Tang et al., 2008a; Tang et al., 2008b; Chen et al., 2008; Zhang et al., 2009; Hu and Ou, 2010; Qi, 2010).

NDQG are generally beneficial in improving the level of glucose metabolism. NDQG can reduce FBG [SMD = −0.18, 95% CI (−0.38, 0.03), *p* = 0.10, *I* ^*2*^ = 71.18%] and HbA1c [SMD = −0.42, 95% CI (−0.86, −0.02), *p* = 0.06, *I* ^*2*^ = 81.64%], but the effect on 2hPG [SMD = −0.04, 95% CI (−0.61, 0.53), *p* = 0.89, *I* ^*2*^ = 84.33%] is not obvious, which suggests that NDQG in the treatment of DKD lower HbA1c by reducing FBG. In 2010, the ADA guidelines for diabetes management included glycated hemoglobin ≥6.5 percent as one of the diagnostic criteria for type 2 diabetes (T2DM) (American Diabetes Associtaion, 2010). HbA1c reflects the average blood glucose level over the past 2–3 months and is an important criterion for assessing control blood glucose. The UK Prospective Diabetes Study has shown that the risk of developing various complications in people with T2DM is strongly related to blood sugar control. For every 1% reduction in HbA1c, the risk of all diabetes-related endpoints was reduced by 21%, the risk of diabetes-related death by 21%, the risk of myocardial infarction by 14%, and the risk of microvascular complications by 37% (Stratton et al., 2000).

Adjuvant treatment with NDQG can reduce TC [SMD = −0.70, 95% CI (−1.01, −0.39), *p* < 0.001, *I* ^*2*^ = 86.74%] and TG [SMD = −0.61, 95% CI (−0.87, −0.36), *p* < 0.001, *I* ^*2*^ = 80.64%], which are the risk factors of DKD. However, due to the small number of included studies on HDL-C [SMD = 0.71, 95% CI (0.02, 1.40), *p* = 0.04, *I* ^*2*^ = 87.43%], it is unclear whether NDQG have an improved effect on HDL-C. Dyslipidemia is a common risk factor for cardiovascular disease, which in turn is the main cause of morbimortality in CKD and T2DM (Hager et al., 2017). Abnormalities in the lipid metabolism promote progression of kidney damage (Kramer et al., 2006). According to our findings, combining NDQG with CT could be a beneficial therapy for DKD patients with abnormal TC and TG metabolisms.

In all patients with CKD, patients with DKD are more likely to develop anemia than those with non-diabetic kidney disease (NDKD) (Ito et al., 2021), so we investigated the effect of NDQG on Hb, and studies have shown that adjuvant treatments with NDQG are not much different from CT in increasing Hb [SMD = 0.11, 95% CI (−0.10, 0.32), *p* = 0.32, *I* ^*2*^ = 0.00]. This is contrary to the conclusion that NDQG can improve anemia caused by kidney disease (Zhang and Lei, 2012). We think there could be two reasons for this: on the one hand, it may be due to NDQG having no benefit on kidney anemia caused by DKD; on the other hand, it may be due to the small number of included literature and the low quality of the literature, so more high-quality literature is needed to explore this indicator.

Chronic microinflammatory states are common in patients with CRF, and inflammatory factors associated with microinflammatory states include Hs-CRP, IL-6, IL-8, and TNF-α (Zhu et al., 2018). We found that NDQG have beneficial effects in reducing Hs-CRP [SMD = −1.00, 95% CI (−1.54, −0.46), *p* < 0.001, *I* ^*2*^ = 86.81%], IL-18 [SMD = −1.25, 95% CI (−1.58, −0.92), *p* < 0.001, *I* ^*2*^ = 0], and TNF-α [SMD = −1.28, 95% CI (−1.64, −0.91), *p* < 0.001, *I* ^*2*^ = 75.73%]. It has been proven that NDQG can have a positive effect on the disease by improving the microinflammatory state of DKD.

Oxidative stress plays an important role in the development of CRF, and studies have shown that reducing oxide production or increasing the body’s antioxidant capacity can improve oxidative stress damage in renal tissue in patients with CRF (Kim and Vaziri, 2010). This analysis found that NDQG mainly improve oxidative stress in DKD by reducing MDA [SMD = −0.88, 95% CI (−1.22, −0.54), *p* < 0.001, *I* ^*2*^ = 66.01%] and AOPP [SMD = −0.92, 95% CI (−1.85, 0.00), *p* < 0.001, *I* ^*2*^ = 90.68%].

Of the 52 included studies, adverse events were mentioned in 24 studies (Lv et al., 2008; Bai and Shi, 2010; Xiao et al., 2011; Zhang et al., 2011; Wang et al., 2012; Gong, 2013; Wang et al., 2013; Li, 2015; Wan, 2015; Li et al., 2016; Wang, 2016; Zhang et al., 2016; Li et al., 2017; Wei and Ruan, 2018; Ding and Zhang, 2019; Liu and Yang, 2020; Chen et al., 2021; He, 2021; Kou, 2021; Lan et al., 2021; Li et al., 2021; Liang et al., 2021; Wu, 2021; Li, 2022), of which adverse events occurred in 19 studies (Lv et al., 2008; Bai and Shi, 2010; Xiao et al., 2011; Wang et al., 2012; Wang et al., 2013; Wan, 2015; Li et al., 2016; Li et al., 2017; Wei and Ruan, 2018; Ding and Zhang, 2019; Liu and Yang, 2020; Chen et al., 2021; He, 2021; Kou, 2021; Lan et al., 2021; Li et al., 2021; Liang et al., 2021; Wu, 2021; Li, 2022). Adverse events were mentioned in the remaining five studies (Zhang et al., 2011; Gong, 2013; Li, 2015; Wang, 2016; Zhang et al., 2016), but no adverse events were observed in either groups. The remaining 28 studies did not report adverse events, suggesting that these researchers did not pay enough attention to adverse events. We also found that adjuvant treatment with NDQG did not increase the incidence of adverse reactions in the control group [SMD = 0.98, 95% CI (0.71, 1.34), *p* = 0.89, *I* ^*2*^ = 1.59%]. Adverse reactions include gastrointestinal reactions, dry mouth, hyperkalemia, hypoglycemia, allergies, dizziness, headache, fatigue, flushing, dry cough, fever, rash, and hypercalcemia, of which gastrointestinal reactions are the most common, appearing 17 times. None of these studies had patients withdrawn from the RCT; however, only two studies (Wang et al., 2013; Li et al., 2016) mentioned that side effects disappeared after NDQG reduction, and the rest of the literature did not treat them or did not mention how to deal with them. These results show that the proper use of NDQG has a high safety profile in clinical use.

However, the heterogeneity of the 22 indicators analyzed in this study was high (*I* ^*2*^ > 50%), which may be caused by different disease and treatment courses.

### 5.2 The main components of NDQG and possible mechanisms

NDQG are a pure Chinese medicine preparation composed of 16 herbs, which have the advantages of being a component and multi-target and have played a unique role in the treatment of DKD. Modern pharmacological studies have found that the main components of NDQG include rhubarb acid, matrine, astragaloside IV, peony glycoside, Salvianic acid A and protocatechin, stilbene glycoside, and salvianolic acid B (Zou and Zhou, 2009; He et al., 2011; Chen, 2015; Zhao, 2017; Zhao et al., 2022). Previous studies have shown that NDQG can reduce the expression level of the pro-inflammatory factor interleukin in the blood of DKD patients, alleviate platelet activation status, and delay renal failure (Liang et al., 2020), and the active ingredients such as isoflavones and peony glycosides can significantly improve kidney function (Zhou et al., 2020). NDQG have been widely used in clinical practice, which can effectively reduce the level of SCr and BUN, stabilize and protect residual kidney function, help discharge accumulated toxins in the body, and reduce the burden on patients’ kidneys (Layton et al., 2020). The total glycoside of white peony in NDQG can effectively improve the immune function of rats and effectively reduce inflammatory response and oxidative stress response (Yan et al., 2018); white urinary lactone I and white urinary lactone III can significantly inhibit the production of TNF-α and inhibit the activity of TNF-αmRNA; atractylenolide I has a stronger inhibitory effect than atractylenolide III (Li et al., 2007); chuanxiongzine can reduce the 24hUTP levels in rats with IgA nephropathy, inhibit the deposition of IgA in the mesangial region, and downregulate TGF-β mRNA in kidney tissue and TGF-β protein levels (Lin et al., 2013); and oxymatrine inhibits renal fibrosis (Chen and Jin, 2014).

In summary, the intrinsic reasons for NDQG to improve kidney function and regulate glycolipid metabolism may be related to factors such as the fact that its active ingredients can inhibit oxidative stress and reduce and regulate inflammatory response.

### 5.3 Possible mechanisms of side effects of NDQG

The mechanism of adverse reactions caused by NDQG is not clear. Among these 16 herbs in NDQG, rhubarb (Dahuang, *Rheum palmatum* L.), Radix Polygoni Multiflori (Heshouwu, *Reynoutria multiflora* (Thunb.) Moldenke), *Pinellia* tuber (Banxia, *Pinellia ternata* (Thunb.) Makino), and Chinese thorowax root (Chaihu, *Bupleurum falcatum* L.) were shown to cause adverse reactions in five studies (Yang et al., 2012; Yang, 2015; Sun et al., 2016; Wei and Liu, 2018; Yang et al., 2023). Among them, rhubarb (Dahuang, *Rheum palmatum* L.) contains anthraquinones, polysaccharides, tannins, and other components which cause liver toxicity, renal toxicity, and gastrointestinal toxicity in normal animals (Wei and Liu, 2018). The main material basis that can cause irritating toxicity in *Pinellia* tuber (Banxia, *Pinellia ternata* (Thunb.) Makino) is calcium oxalate needle crystals formed by the combination of calcium oxalate and protein (Yang et al., 2023), which will stimulate the mucosa to cause cell damage, release inflammatory mediators, and produce pinprick-like tingling; however, ginger–Pinellia tuber (Banxia, *Pinellia ternata* (Thunb.) Makino) is used in NDQG, and the Pinellia tuber (Banxia, *Pinellia ternata* (Thunb.) Makino) after concocting with ginger becomes soothing and spicy warm, which enhances its anti-nausea effect and can also play a role in strengthening the stomach. The main component of Radix Polygoni Multiflori (Heshouwu, *Reynoutria multiflora* (Thunb.) Moldenke) is stilbene, and it may cause skin itching, sweating, fever, chills, general weakness, dry mouth, and dizziness clinically (Yang, 2015). The main active ingredients of Chinese thorowax toot (Chaihu, *Bupleurum falcatum* L.) mainly include saponins, volatile oils, flavonoids, and polysaccharides (Yang et al., 2012), and Chinese thorowax toot (Chaihu, *Bupleurum falcatum* L.) shows hepatotoxicity and nephrotic properties in rats (Sun et al., 2016). We suspect that the adverse effects caused by NDQG may be caused by the aforementioned drugs. The side effects of NDQG still need further pharmacological and toxicological studies to be explored, and the mechanism of the synergistic effect has not been revealed completely and requires more investigations.

### 5.4 Limitations of the study

Although we efficiently carried out the standard analytical methods, this study has some limitations. First, according to Figure 2C, there was asymmetry on both sides and publication bias, possibly due to the poor methodological quality of the included studies, unclear randomization methods in most of the 52 included studies, lack of blinding, and failure to report allocation concealment. Second, some studies did not fully report the study characteristics such as disease course and treatment course, so the choice of heterogeneity analysis methods and the exploration of dominant populations were limited. Third, the included studies were single-center, small-sample studies and may not be representative. Fourth, the included studies were all Chinese literature, which may have ethnic and regional limitations.

## 6 Conclusion

In summary, we found that combination treatment with NDQG has more advantages than CT alone in DKD treatment, which could improve clinical efficiency, kidney function, blood glucose level, blood lipid level, inflammatory factors, and oxidative stress indicators. At the same time, we found that NDQG are also relatively safe. However, more high-quality studies are needed to provide more reliable evidence for clinical use.

## Figures and Tables

**Table:** 

**24UTP**	24-h urine protein quantification
**2hPG**	2-h postprandial blood glucose
**AOPP**	Advanced oxidation protein products
**BUN**	Blood urea nitrogen
**CCr**	Creatinine clearance rate
**CI**	Confidence interval
**CNKI**	China National Knowledge Infrastructure
**CRF**	Chronic renal failure
**CT**	Conventional treatment
**Cys-C**	Cystatin-c
**DKD**	Diabetic kidney disease
**DN**	Diabetic nephropathy
**eGFR**	Estimated glomerular filtration rate
**ESRF**	End-stage renal failure
**FBG**	Fasting blood glucose
**Hb**	Hemoglobin
**HbA1c**	Glycosylated hemoglobin
**HDL-C**	High-density lipid-cholesterol
**Hs-CRP**	High-sensitivity C-reactive protein
**IL-18**	Interleukin-18
**MD**	Mean difference
**MDA**	Malondialdehyde
**NDQG**	Niaoduqing Granules
**PRISMA**	Preferred Reporting Items for Systematic Review and Meta-Analysis;
**PROSPERO**	International Prospective Register of Systematic Reviews
**RCT**	Randomized controlled trial
**RCTs**	Randomized controlled trials
**RR**	Relative risk
**SCr**	Serum creatinine concentration
**SMD**	Standardized mean difference
**SOD**	Superoxide dismutase
**T2DM**	Type 2 diabetes mellitus
**TC**	Total cholesterol
**TCM**	Traditional Chinese Medicine
**TCMSP**	Traditional Chinese Medicine Systems Pharmacology Database and Analysis Platform
**TG**	Triglyceride
**TGF-β1**	Transforming growth factor-β1
**TNF-α**	Tumor necrosis factor-α
**UA**	Uric acid
**UAER**	Urinary albumin excretion rate
**VIP**	China Science and Technology Journal Database

## Data Availability

The original contributions presented in the study are included in the article/Supplementary Materials, further inquiries can be directed to the corresponding author.
